# Synergistic effect of sulfonation followed by precipitation of amorphous calcium phosphate on the bone-bonding strength of carbon fiber reinforced polyetheretherketone

**DOI:** 10.1038/s41598-023-28701-1

**Published:** 2023-01-25

**Authors:** Yusuke Takaoka, Shunsuke Fujibayashi, Takeshi Yabutsuka, Yuya Yamane, Chihiro Ishizaki, Koji Goto, Bungo Otsuki, Toshiyuki Kawai, Takayoshi Shimizu, Yaichiro Okuzu, Kazutaka Masamoto, Yu Shimizu, Makoto Hayashi, Norimasa Ikeda, Shuichi Matsuda

**Affiliations:** 1 Department of Orthopedic Surgery, Kyoto University Graduate School of Medicine, Kyoto, Japan; 2 Department of Fundamental Energy Science, Graduate School of Energy Science, Kyoto University, Kyoto, Japan

**Keywords:** Biomedical materials, Implants

## Abstract

Sulfonation and applications of amorphous calcium phosphate are known to make polyetheretherketone (PEEK) bioactive. Sulfonation followed by precipitation of amorphous calcium phosphate (AN-treatment) may provide PEEK with further bone-bonding strength. Herein, we prepared a carbon-fiber-reinforced PEEK (CPEEK) with similar tensile strength to cortical bone and a CPEEK subjected to AN-treatment (CPEEK-AN). The effect of AN-treatment on the bone-bonding strength generated at the interface between the rabbit’s tibia and a base material was investigated using a detaching test at two time-points (4 and 8 weeks). At 4 weeks, the strength of CPEEK-AN was significantly higher than that of CPEEK due to the direct bonding between the interfaces. Between 4 and 8 weeks, the different bone forming processes showed that, with CPEEK-AN, bone consolidation was achieved, thus improving bone-bonding strength. In contrast, with CPEEK, a new bone was absorbed mainly on the interface, leading to poor strength. These observations were supported by an in vitro study, which showed that pre-osteoblast on CPEEK-AN caused earlier maturation and mineralization of the extracellular matrix than on CPEEK. Consequently, AN-treatment, comprising a combination of two efficient treatments, generated a synergetic effect on the bonding strength of CPEEK.

## Introduction

Polyetheretherketone (PEEK) is a high-performance engineering plastic with exceptional resistance to chemicals, wear, and fatigue. In addition, because of its mechanical properties and biocompatibility, it is used as an orthopedic implant^1–3^. Radiolucency, one of its most attractive characteristics, enables clear interpretation of postoperative medical images^2,3^. Furthermore, according to the circumstances, PEEK can change its tensile strength to the desired strength by carbon-fiber reinforcement, and the tensile strength of 50% carbon-fiber-reinforced PEEK (CPEEK) is about 120 MPa and close to that of cortical bones^4–6^. Therefore, it potentially resolves the problem caused by gaps in tensile strength between implants and bones, for example, stress shielding^7–9^, (often seen when metallic implants are used), and the weakness of pure PEEK material.

Because of its chemical inertness, PEEK is not bioactive. Therefore, various treatments have been used to achieve osseointegration with PEEK^9–12^. Among them, sulfonation^9,11,13–16^ and applications of amorphous calcium phosphate (ACP)^17–19^ are popular treatments for gaining bone-bonding strength in PEEK implants. Sulfonation, acid-etching by immersion in sulfuric acid, changes the surface topography and gives proton conductivity to the benzene rings of PEEK^13,20^ for chemical bonding with other compounds, including ACP^17^. Despite the advantage of sulfuric acid immersion in changing the surface topography, including pore size, it may be a disadvantage for this treatment. If the immersion time is too long, it harms the human body because of the residual sulfuric acid on the substrates^13,14,21^. Additionally, it damages PEEK itself and weakens its material strength^14,21^.

Alternatively, ACP is used as coatings and cement for orthopedic and dental applications^17^. ACP is an intermediate phase that precipitates from a highly supersaturated calcium phosphate solution and converts easily to a stable crystalline phase. ACP is already known to precipitate on the surface of materials in solution with Ca^+^ and PO_4_^3−^ by increasing the pH and temperature of the solution^17,22^. It has better conductivity and biodegradability than hydroxyapatite and tricalcium phosphate^18^. In this way, ACP is expected to not only overcome the major drawbacks of the excellent method, sulfuric acid treatment, but also extend its advantages. In this study, we focused on both treatments and tried to combine them for a synergetic effect. AN-treatment involves short-time sulfonation and glow discharging followed by precipitation of ACP produced by immersion in modified simulated body solution (SBF), dubbed “apatite nuclei”, developed for better bone-bonding strength^23–25^. Modified SBF identified in previous studies to search for the ideal SBF for better apatite deposition on CPEEK^23^.

When implants are embedded in a living body, fibrous tissues form on its surface, interfering with the direct bonding with the bone^9,12^. This encapsulation, sometimes seen as a radiolucent line in the post-operative X-ray, results in the failure of implantation caused by aseptic loosening^26^. Furthermore, thick layers of fibrous tissues are unsuitable for weight translation^26^. Additionally, this encapsulation site can become the site of inflammatory responses to fine particles caused by the wear of the implant, for example, the bearing surface of artificial joints^27^. Ideally, the integration without encapsulation between the base material and new bone will improve bone-bonding strength and resolve these problems.

Bone-bonding strength is essential for osseointegration in vivo to get rigid fixation during functional loading. This is achieved by ossification, based on the delicate balance between bone formation and resorption, which changes with time^28^ and requires two time-points evaluations. Therefore, this study aimed to measure the interfacial bone-bonding strength at two time-points (4 and 8 weeks after implantation), with CPEEK and CPEEK-AN, to evaluate the effect of AN-treatment on the PEEK interface. Furthermore, we investigated the factors in an in vitro study that may influence the differences in bone-bonding strength, with and without the treatment.

## Results

### Substrates

#### Surface characteristics

**Figure 1:**
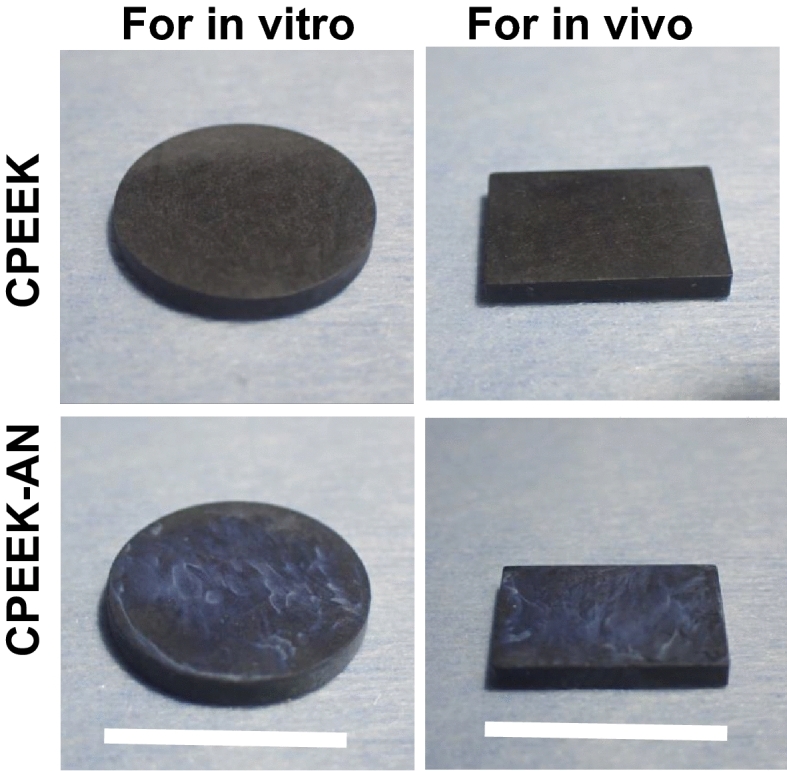
Photographs of the substrates (CPEEK, CPEEK-AN) for in vivo and in vitro study. White bar indicates 15 mm.

**Figure 2:**
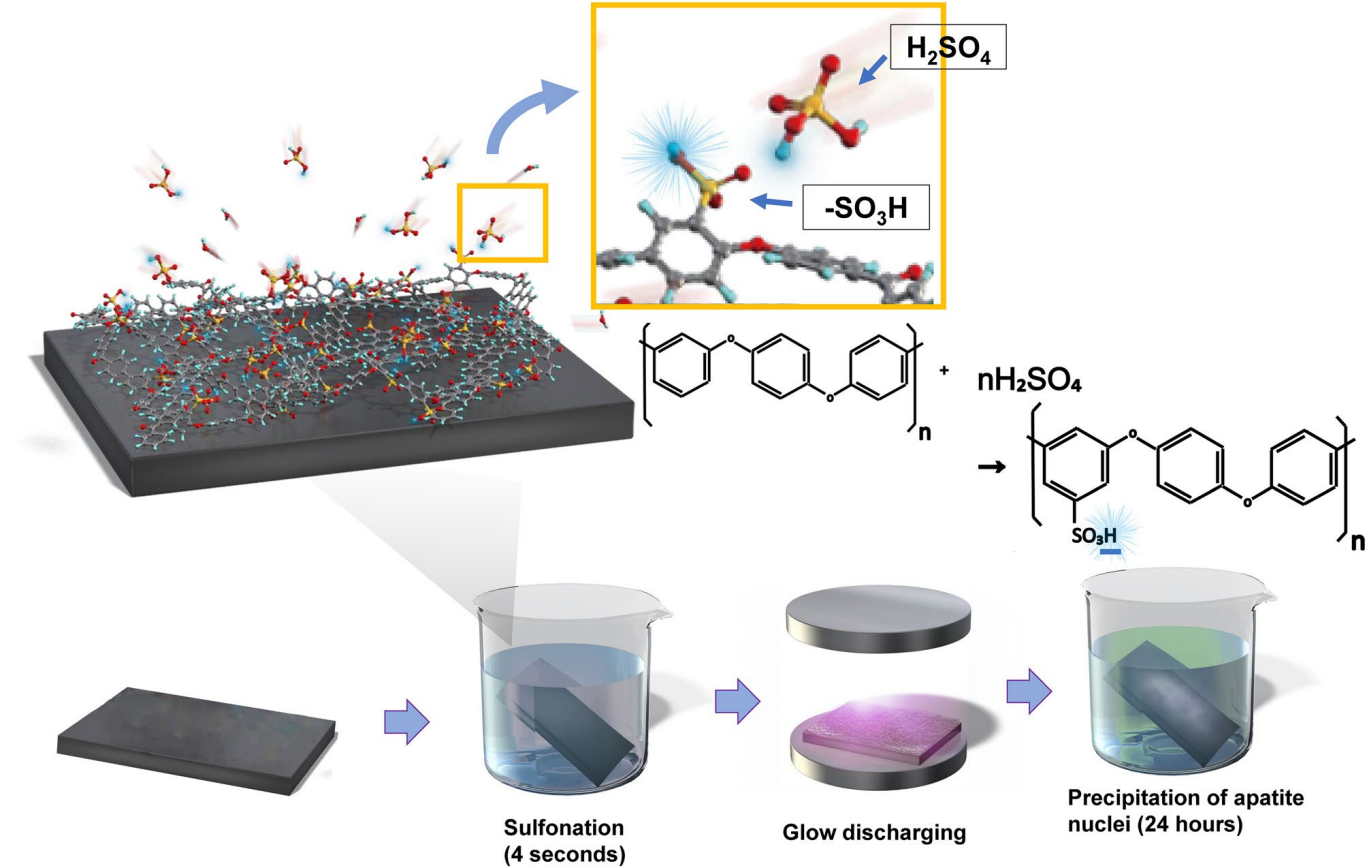
Schematic diagram of the fabrication process for CPEEK-AN, and its magnification, as well as the reaction equation for sulfonation. Emphasis of H in –SO_3_H expresses the proton conductivity.

**Figure 3:**
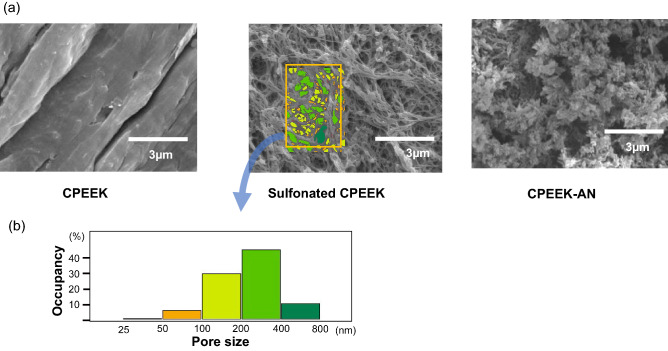
(**a**) SEM photographs showing the surface of CPEEK, sulfonated PEEK, and CPEEK-AN. (**b**) Characterization of pores formed on the sulfonated PEEK.

**Figure 4:**
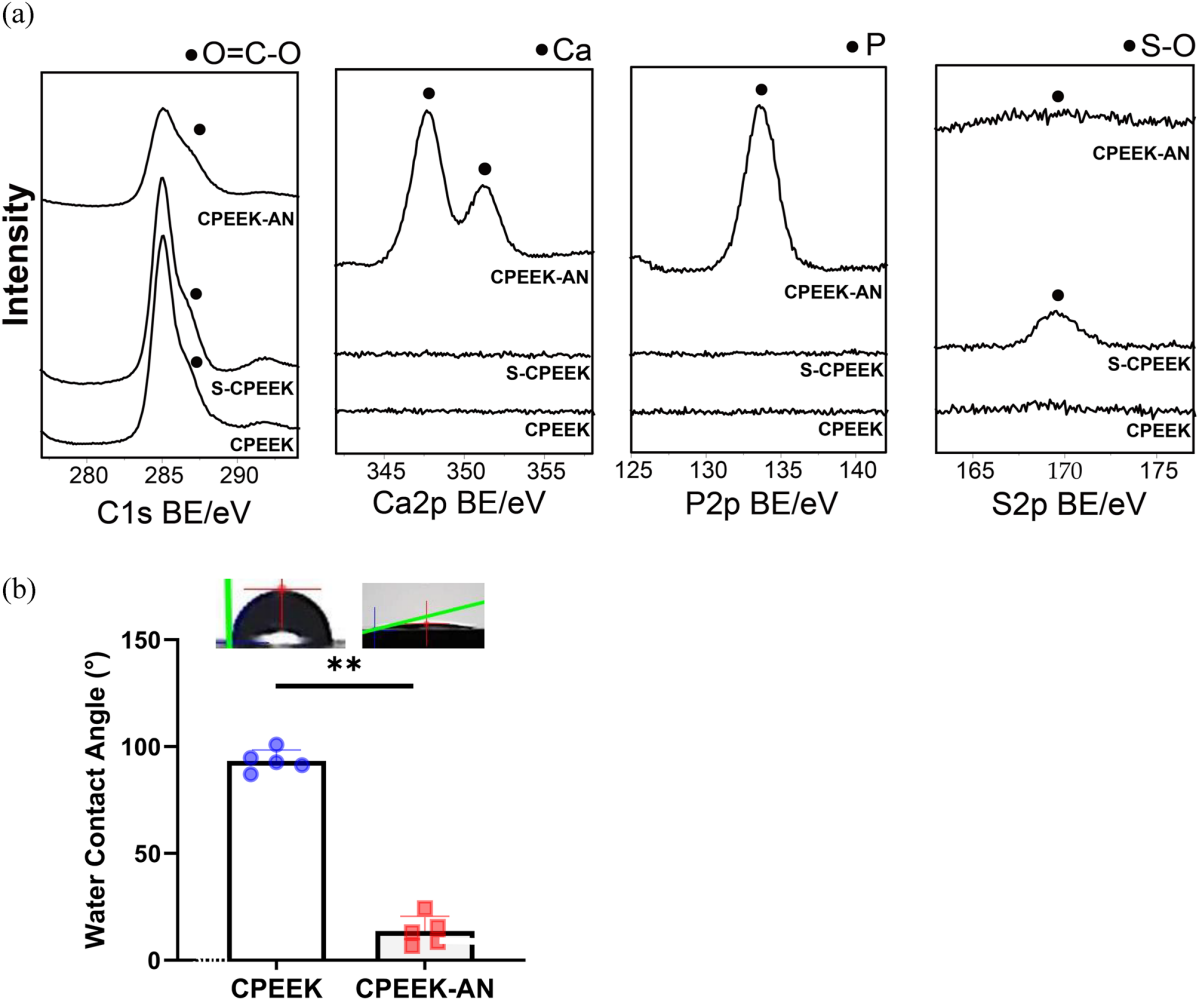
Surface characteristics. (**a**) XPS narrow spectra around the binding energy of C1s, Ca2p, P2p, and S2p on the surface of CPEEK, sulfonated PEEK (S-CPEEK), and CPEEK-AN. (**b**) Water contact angle measured with a contact angle meter. The symbol ‘**” indicates p < 0.01.

There was no obvious difference between CPEEK and CPEEK-AN substrates; however, that of CPEEK-AN looked slightly white (Fig. 1). A sequential procedure, including immersion in sulfuric acid twice for 4 s, exposing glow disposing, and immersion in modified SBF (Table 1) for 24 h changed the surface (Fig. 2). From the scanning electron microscopy (SEM) observation (Fig. 3a), the CPEEK surface was changed to a complicated porous structure by sulfonation. This porous structure possessed various shapes and diameters to 800 nm, as shown in Fig. 3. The surface after AN-treatment was almost covered with the precipitate of apatite nuclei (Fig. 3a); this was confirmed by the X-ray photoelectron spectroscopy (XPS) profile, as shown in Fig. 4a. Although CPEEK-AN showed peaks intensity in Ca2p and P2p derived from calcium (Ca) and phosphorus (P), respectively, no peaks were observed for CPEEK other than C1s derived from O=C–O. Additionally, the peak derived from S–O, which expresses the presence of sulfuric acid, observed in sulfonated CPEEK was weakened in CPEEK-AN. The water contact angle showed that this treatment improved wettability (Fig. 4b). A detailed evaluation of the properties of these materials has been reported in previous studies.

**Table 1:** Amounts of dissolved reagent in the preparation of 1 dm^3^ SBF and modified-SBF.

Reagent	Dissolved amount or volume in 1 dm^3^	
	SBF	Modified SBF
NaCl	137 mmol·dm^−3^	–
NaHCO_3_	4.17 mmol·dm^−3^	–
KCl	3.00 mmol·dm^−3^	–
K_2_HPO_4_·3H_2_O	1.31 mmol·dm^−3^	1.31 mmol·dm^−3^
MgCl_2_·6H_2_O	1.72 mmol·dm^−3^	1.72 mmol·dm^−3^
1 mol·dm^−3^ HCl	35 cm^3^·dm^−3^	35 cm^3^·dm^−3^
CaCl_2_	2.50 mmol·dm^−3^	2.50 mmol·dm^−3^
Na_2_SO_4_	0.50 mmol·dm^−3^	–

#### Evaluation of the apatite-forming ability

After soaking in SBF^29^ (Table 1) for 1 day, apatite formation was confirmed on the surface of CPEEK-AN, whereas almost no deposition was observed on other substrates (Fig. S1). This study was performed before the in vivo and in vitro experiments; we compared CPEEK and CPEEK-AN based on the results. The sulfonated CPEEK was not included in subsequent experiments.

### In vivo study

#### Bone-bonding strength evaluation by the detaching test

**Figure 5:**
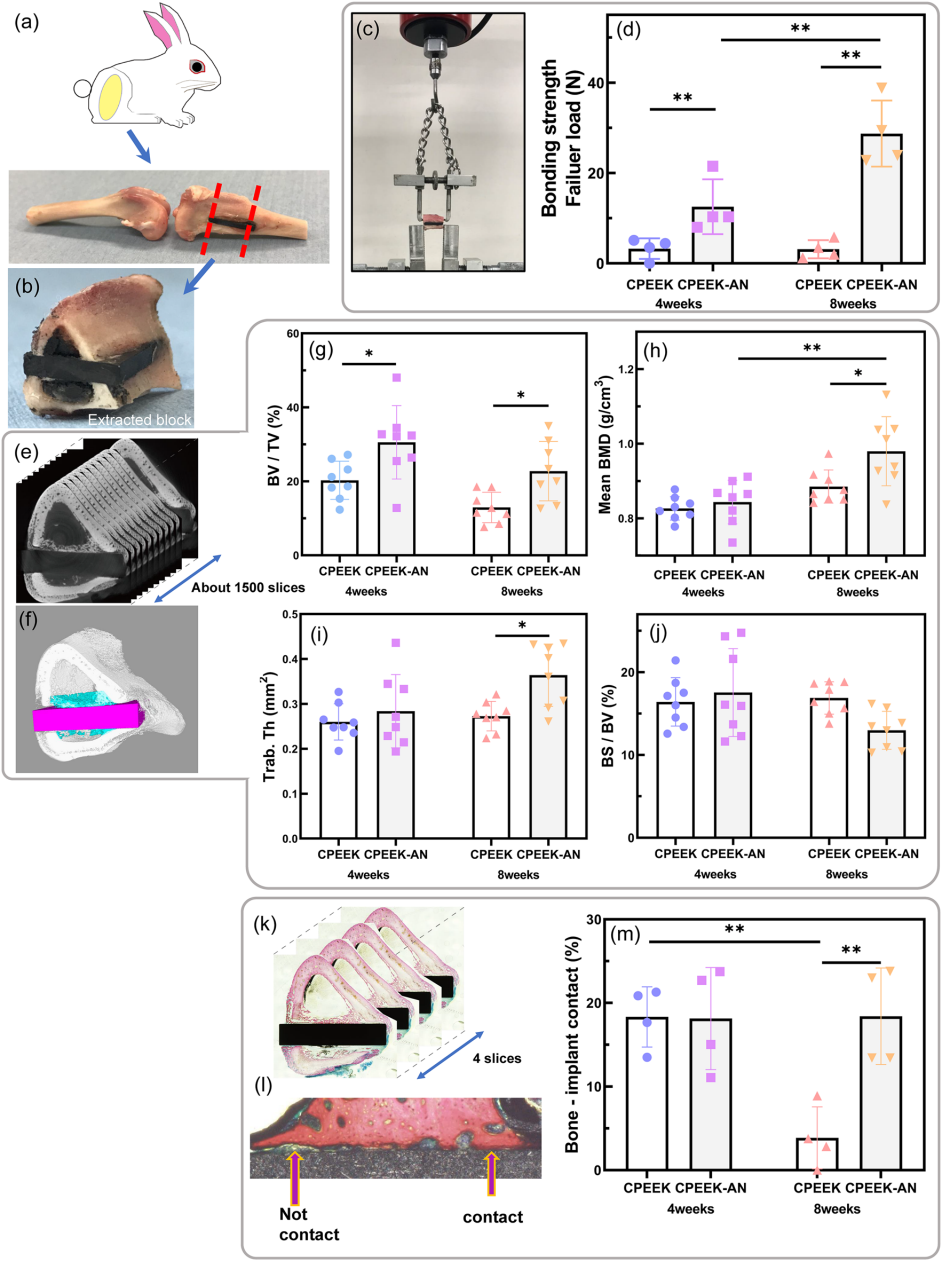
In vivo study. (**a**) Schematic illustration of a Japanese white rabbit and the removed femur and tibia bones which includes the substrate after sacrifice. (**b**) The extracted block, cut from the tibia bone, including a substrate. (**c**) The apparatus for the detaching test. (**d,e**) Reconstruction of a 3D model using around 1500 μ-CT image slices taken from the extracted block. 3D-model shows new bone (blue), within 1 mm on the surface of the substrate (purple). (**f**) An illustration depicting a region in contact vs the one not in contact. (**g**) Four histological slices taken from the block. (**h**) Bonding strength. (**i**) Bone volume/total volume (BV/TV, %), (**j**) mean bone mineral density (Mean BMD, g cm^−3^). (**k**) Trabecular thickness (Trab. Th, mm^2^), and (**l**) bone surface/bone volume (BS/BV, %), based on (**d,e**) evaluation. (**m**) Bone-implant contact (BIC) ratio (%), based (**f**) quantification. The symbol ‘‘*” indicates p < 0.05 and ‘‘**” indicates p < 0.01.

The extracted blocks were obtained from the femur of rabbits to evaluate the interfacial bone-bonding strength between bones and substrates with the apparatus (Fig. 5a–c). As shown in Fig. 5d the average failure loads obtained from each group (CPEEK in week 4, CPEEK-AN in week 4, CPEEK in week 8, and CPEEK-AN in week 8) were 3.25 ± 2.3N, 12.5 ± 6.1 N, 3.13 ± 2.0 N, and 28.7 ± 7.3 N, respectively. Four weeks after implantation, the AN-treatment significantly improved the bonding strength of the CPEEK. Furthermore, the strength of CPEEK-AN improved significantly between 4 and 8 weeks, whereas that of CPEEK was insignificant during this term.

#### Radiological evaluation with μ-CT

New bone, defined as the bone in the intramedullary area within 1 mm width from the surface of the base material, was assessed with µ-CT (Fig. 5e,f). Bone volume/total volume (BV/TV) and consolidation indicators, including bone mineral density (BMD), trabecular thickness (Trab.Th), and bone surface/bone volume (BS/BV), were calculated. Trab.Th was thicker with decreased BS/BV (Fig. S2). At 4 weeks, BV/TV for CPEEK-AN was significantly higher than that for CPEEK (CPEEK 20.3 ± 5.2 N, CPEEK-AN 30.6 ± 9.9 N); however, other consolidation indicators showed no significant difference between the two (Fig. 5g–j). Interestingly, BV/TV at 8 weeks for both substrates decreased as time passed (Fig. 5g); however, the value for CPEEK-AN was still higher than that for CPEEK (CPEEK 12.9 ± 4.1 N, CPEEK-AN 22.8 ± 8.0 N). In contrast, other consolidation indicators for CPEEK-AN improved at 8 weeks, and BMD and Trab.Th with and without treatments showed a significant difference.

#### Histological evaluation

Four slices from each extracted block were used to assess the bone-implant contact (BIC) ratio determined by the contact between bone and substrates (Fig. 5k,l). BIC ratios were approximately the same at 4 weeks for both groups, as shown in Fig. 5m. In contrast, at 8 weeks, the BIC ratio of CPEEK changed significantly, and that of CPEEK-AN was unchanged.

#### The interfacial evaluation between new bone and substrates

**Figure 6:**
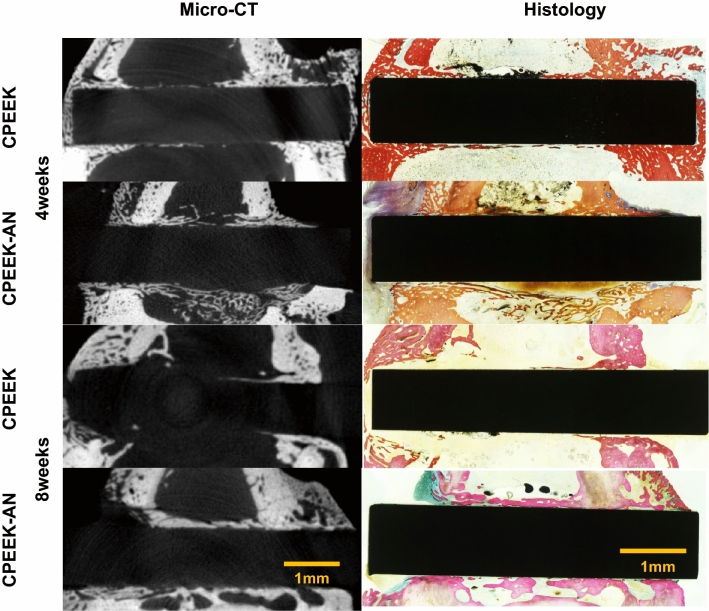
Interfacial evaluation1. Micro-CT images and the corresponding histological images of the specimen at 4 and 8 weeks after surgeries.

**Figure 7:**
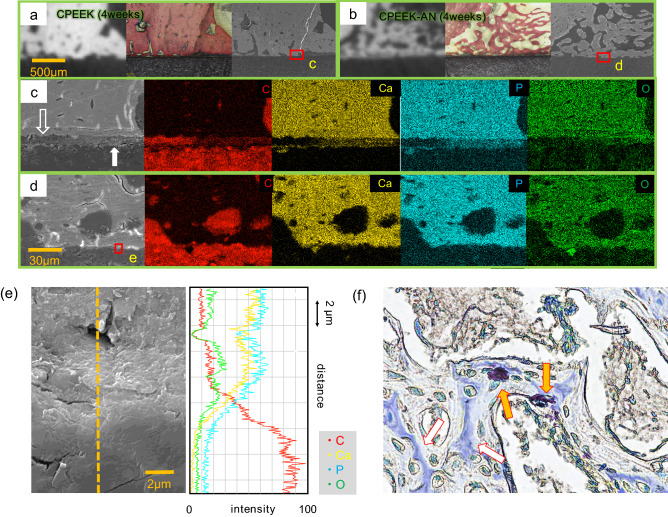
Interfacial evaluation2. Corresponding CT, histological, and SEM images of samples taken from a 4-week-old rabbit (**a**) with CPEEK and (**b**) with CPEEK-AN. (**c**) Magnified image of (**a**) using SEM and matched EDX mapping analysis of the composite. The hollow arrow points to the gap between the implant and the new bone. The solid white arrow indicates the middle progress during ossification. (**d**) Magnified image of (**b**) using SEM and matched EDX mapping analysis of the composite. (**e**) The line analysis for (**e**) which includes the interface between CPEEK and the new bone. (**f**) A section of the histological specimen of CPEEK-AN at 4 weeks co-stained with TRAP and ALP; this image illustrates a typical woven bone. The orange arrows indicate the TRAP positive cells and the hollow arrows indicate an ALP positive part of the new bone at the 4-week timepoint.

µ-CT images and the almost corresponding histological images were prepared for overall evaluation of the interface between the bone and each substrate (Fig. 6). At 4 weeks, both CPEEK and CPEEK-AN formed woven bone. However, CPEEK-AN exhibited more woven bone far from substrates than CPEEK. At 8 weeks, the woven bone for CPEEK-AN matured, whereas that for CPEEK disappeared. To further understand whether integration had been achieved, the images with three devices (μ-CT, histological assessment, and SEM) were obtained with higher magnification, showing different insights into the bone-implant interfaces (Fig. 7a,b). Under high magnification with SEM, we observed a gap of < 10 µm, caused by the absence of integration, in the sample for CPEEK (Fig. 7c); however, this gap was absent for CPEEK-AN (Fig. 7d). Energy-dispersive X-ray (EDX) analysis showed two patterns of this gap implying the absence of integration. One is the gap with no Ca or P, and the other is the layer with properties of carbon, Ca, and P seen as a residual apatite nuclear or supposedly middle progress during ossification (Fig. 7c). “Conversely, the line analysis (Fig. 7e) suggested that no clear gap was observed between CPEEK and newly formed bone”.

#### Tartrate-resistant acid phosphatase (TRAP) and alkaline phosphatase staining (ALP) co-staining assay for new bones

The presence of TRAP-positive cells indicated that bone resorption was proceeding. Newly formed bone on CPEEK-AN 4 weeks after implantation, which was partly stained with ALP, possessed osteocytes in each bone lacunae, and TRAP-positive cells were observed on them (Fig. 7f). Similarly, this was observed on CPEEK, and osteoclastic bone resorption for both substrates started even at 4 weeks.

### In vitro study

#### Cell adhesion on substrates

**Figure 8:**
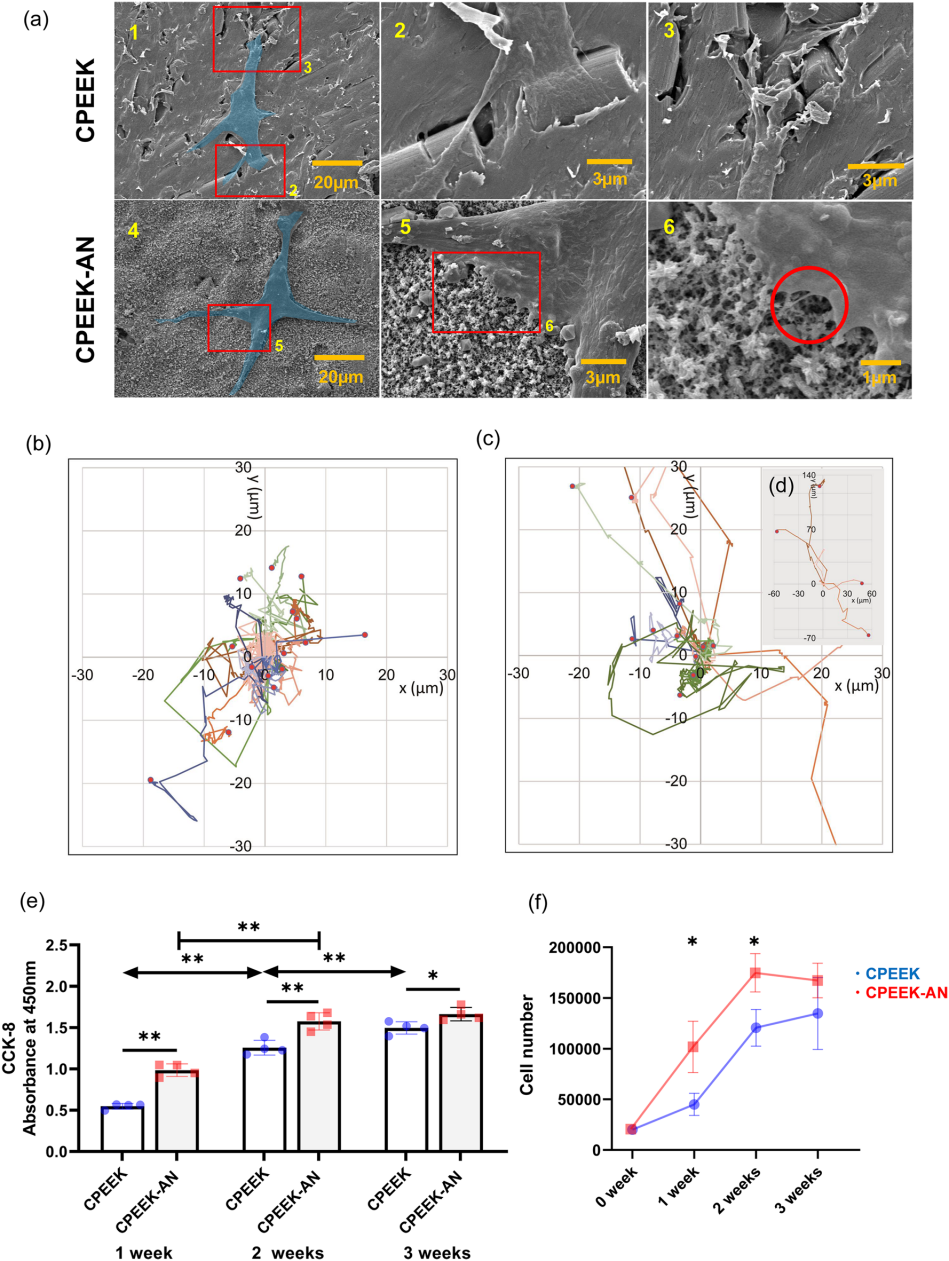
In vitro study1. (**a**) MC3T3-E1 cell (blue) attachment on each substrate after the 24 h incubation, observed using SEM. (a2, 3) and (a5, 6) are magnifications of (a1) and (a4), respectively. (**b**) The motion of stained cells on CPEEK, calculated with time-lapse imaging. This shows the motion with respect to the cells’ first position (X = 0, Y = 0) on the XY coordinate. The red dots indicate their final point at 24 h. (**c**) The plot depicting the XY coordinates for the motion of stained cells on CPEEK-AN. (**d**) The XY coordinates for four cells that moved beyond the XY coordinates of the plot in (**c**). (**e**) Quantification of the CCK-8 assay (absorbance at 450 nm). (**f**) The plot depicting cell numbers (manual counting).

At first glance, the cell morphology of mouse pre-osteoblasts MC3T3-E1 on CPEEK and CPEEK-AN appeared identical (Fig. 8a). However, for CPEEK, the cells spread their cytoplasmic protrusions to the lumpy portion caused by incorporated carbon fiber. For a while, the cells on CPEEK-AN had numerous filamentous pseudopodia from lamellipodia connected directly to apatite nuclei.

#### Cell migration after seeding

MC3T3-E1 cells migrated for 24 h on CPEEK-AN, and their motions on CPEEK-AN were more active than on CPEEK, as shown in Fig. 8b–d. However, the total migration lengths on CPEEK and CPEEK-AN were almost equal (Fig. S3). In addition, the maximum velocity on CPEEK-AN was larger than on CPEEK; however, the difference was insignificant (P value = 0.24). (Fig. S4). Conversely, the CPEEK- AN cells at the end were significantly farther from the first position than CPEEK cells, as shown in Fig. 8b–d and Fig. S5.

#### Cell proliferation on substrates

The proliferation of MC3T3-E1 cells after 1, 2, and 3 weeks of seeding were evaluated with the CCK-8 assay spectrometrically and cell counting by stained cell nuclei number (Fig. 8e,f). The results of both assays were similar and showed a significantly large number of CPEEK-AN cells at every time point compared to CPEEK cells. Particularly, 1 week after culturing, approximately twice the number of CPEEK-AN cells were observed. Until 2 weeks, the cell proliferation on CPEEK-AN was rapid; however, it suddenly slowed down after 2 weeks. In contrast, cells on CPEEK gradually proliferated after 1 week and kept growing until 3 weeks.

#### ALP activity

**Figure 9:**
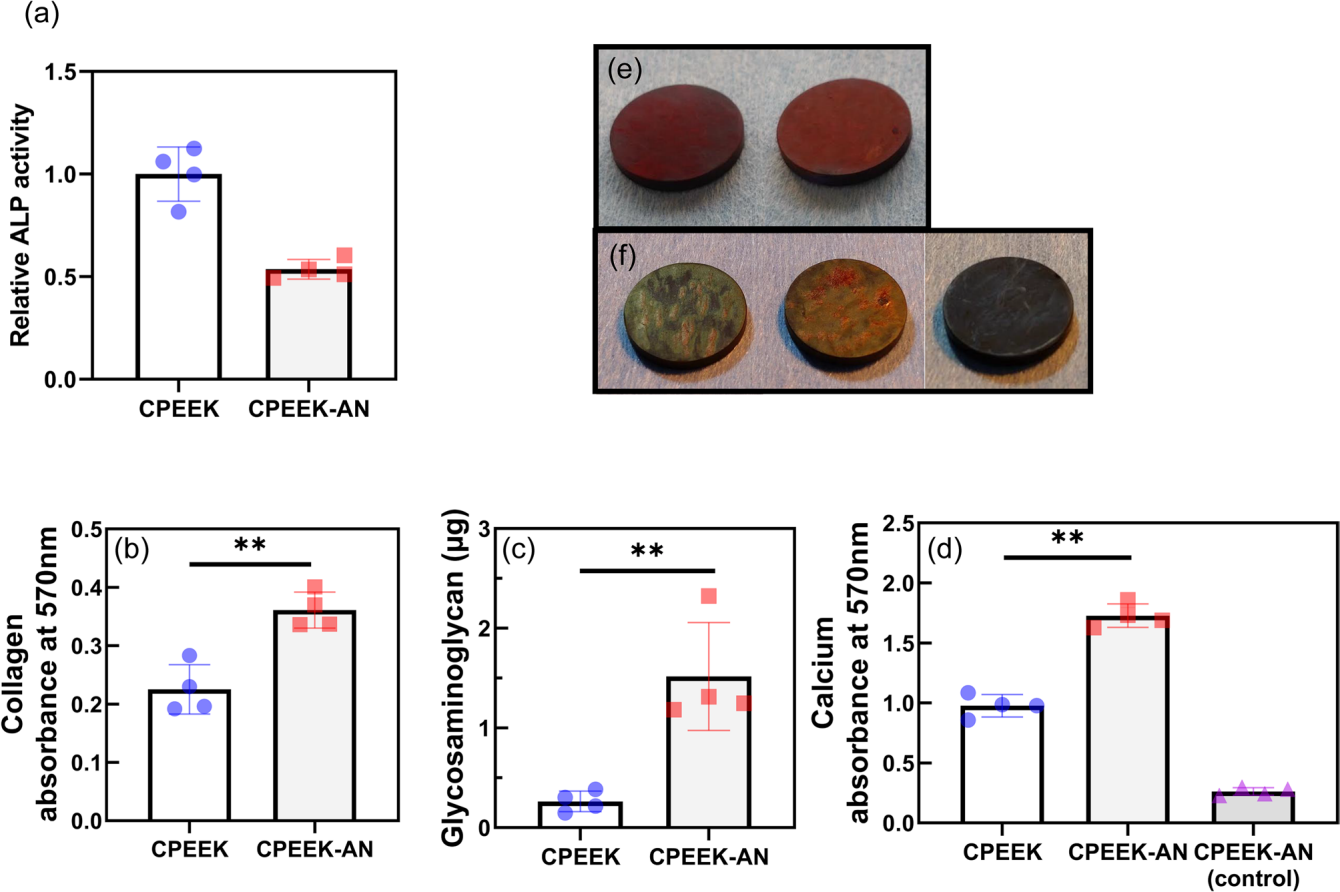
In vitro study2. (**a**) The normalized ALP activity on each substrate. Plots depicting quantifications for (**b**) collagen secretion (absorbance at 570 nm), (**c**) glycosaminoglycan secretion (µg), and (**d**) calcium deposition (570 nm). Gross appearance of (**e**) Sirius red staining for CPEEK (left) and CPEEK-AN (right), and (**f**) alizarin red staining, for CPEEK (left), CPEEK-AN (middle), and control for CPEEK-AN without cells (right). *p < 0.05, **p < 0.01.

The ALP activity was significantly higher for CPEEK than for the normalized total amount of proteins (Fig. 9a). At 3 weeks after seeding, intracellular ALP activities on CPEEK were about twice as high as those on CPEEK-AN.

#### Evaluation of extracellular matrix (ECM) maturation

MC3T3-E1 cells secreted more collagen and glycosaminoglycan (GAG) and deposited more Ca on CPEEK-AN than on CPEEK (Fig. 9b–d). The surface of CPEEK-AN was completely covered with collagen fibers compared to that of CPEEK (Fig. 9e). GAG was secreted five times more for CPEEK-AN than for CPEEK. Additionally, CPEEK-AN demonstrated significant mineralization at 3 weeks, with the Ca nodules plumper than those found on CPEEK (Fig. 9f).

## Discussion

Bone-bonding strength was improved by the AN-treatment, which comprises a combination of sulfonation and ACP application. In addition, based on SEM observation, CPEEK and the new bone were integrated, and the bonding was strengthened over time.

Sulfonation treatment for PEEK is widely used to improve osteoconductivity^11,15^. However, prolonged immersion of PEEK in sulfuric acid results in residual sulfuric acid on the substrates^13,14,21^, which changes its mechanical property^14,21^. In our study, the immersion time is totally 4 s, the shortest time among all studies conducted thus far, although performing this process manually can lead to time reproducibility problems. Consequently, the residual sulfuric acid accumulation was significantly suppressed, as shown in Fig. 4a. in addition, there were a few structural changes; the size of the formed pore was < 1 μm (Fig. 3b). Therefore, the effect on the substrate was considered small. Furthermore, apatite nuclei were precipitated in a time- and cost-effective manner, taking advantage of perfluorosulfonates generated by sulfonation. Notably, the sulfonated CPEEK surface is negatively charged, and positively charged ions, including Ca^2+^, are likely incorporated while small amount of other ions, including Mg^+^, are included. In addition, it has been shown that glow discharging improves hydrophilicity, and increases the ACP deposition^23^. Consequently, ACP was efficiently precipitated. TF-XRD profile and FT-IR spectrum in the previous study showed that this ACP, with extremely low crystallinity, has good apatite-forming ability for only one day in SBF^23–25^. Additionally, the adhesive strength of these apatite nuclei against the substrates was strengthened by the small porous structure produced by sulfonation^25^. This way, two efficient treatments were combined for a synergetic effect.

Bone-bonding strength is essential for clinical use in a rigorous loading environment. Hench et al. insisted that three types of bonding could occur at the interface: mechanical, physical, and chemical^30^. Among them, previous studies indicated that mechanical strength was the dominant factor^12,31^, produced by 300 to 1100 µm-scale porous topology^12,14,15,31,32^. Compared to nano- and micro-scale surface features (example, < 1-µm scale porous or rough), larger-scale (example, 300 to 1100 µm-scale porous) features facilitate bone ingrowth into the cavity, and large volumes of bone result in greater interlocking^33,34^. However, osseointegration attained through this mechanism makes it unclear whether the strength caused by direct bonding is achieved. In this study, the relative planner substrate enabled the evaluation of direct bonding strength without an interlocking mechanism. Thus, direct bonding was achieved, estimated through the SEM observation. We observed gradual composition change in the treated interface without a gap; this bonding improved the bone-bonding strength for CPEEK-AN at 4 weeks. In the early phase, bone consolidation indicators, mineralization, and BIC for both substrates were not significantly different except for BV/TV. Therefore, attaining direct bonding was likely the only factor for the differences in strength, considering that part of the woven bone was far from the interface (Fig. 6) and probably did not contribute to the bonding strength at this phase. Furthermore, direct bonding has another positive effect on the interface. The gap between CPEEK and the new bone could be intruded with fibrous tissue, where inflammatory cells, activated by the fine particle derived from the implant, induce a negative effect, such as osteolysis^26,27^. Direct bonding possibly exclude such a site.

Bone repair after implantation is based on the balance of bone formation by osteoblast and resorption by osteoclast^28,35^. The different transitions of bone-bonding strength for CPEEK and CPEEK-AN between 4 and 8 weeks were possibly caused by the different balances of the ossification process. When the CPEEK surface was not bioactive, the trend towards bone resorption was superior to bone formation because of the lack of AN-treatment. As shown in Fig. 6, the new bone at 4 weeks on CPEEK (osteoid rapidly produced by osteoblasts in response to bone damage)^36,37^, was resorbed mainly on the substrate’s surface at 8 weeks. Additionally, this is explained by the regressive tendency of BV/TV (Fig. 5g) and BIC (Fig. 5m). In contrast, the young bones highly porous woven structure on CPEEK-AN changed to a consolidated lamellar bone structure between 4 and 8 weeks (Fig. 6) while keeping their BV on the surface. Cells related to osteoclastogenesis reportedly take 2 weeks to be active^38^. Figure 7f shows that TRAP-positive cells were observed at 4 weeks, irrespective of the bioactivity of substrates. Accordingly, the trend of bone formation on the CPEEK-AN was superior to that of bone resorption, contradicting the trend on CPEEK. Interestingly, even on CPEEK-AN, BV decreased during this term. This can be explained by the theory that new bone changes to adapt to the subjected stress^39–41^ and does not contribute to the load transmission to reduce BV. Furthermore, an increase in new BV leads to increased bone-bonding strength, especially when a large-scale porous structure is used^12,31^. However, this study showed that the changing BV did not necessarily correspond with bone-bonding strength when the planner substrates were used.

Moreover, the in vitro study showed a better ossification process on the surface with AN-treatment, supporting the in vivo study. Previous studies showed that pre-osteoblast cells, including MC3T3-E1 cells, need scaffolding to attach to the surface by their pseudopodium^42,43^. Additionally, they were known to migrate or transform differently depending on the surface characteristics^43,44^. Accordingly, it was revealed that proper scaffolding was suitable for their attachment and viability, proliferation, and osteogenic differentiation^45^. This study showed that the filamentous pseudopodia from lamellipodia of CPEEK-AN cells were directly attached to the apatite nuclei; however, the CPEEK cells aimed for the lumpy portion. Contrary to expectations, time-lapse imaging (Fig. 8b–d) indicated that substrates did not significantly affect their total migration length or maximum velocity; however, when compared to the CPEEK cells, the CPEEK-AN cells were located farther from the starting point. It is widely known that, after attachment to a surface, MC3T3-E1 cells proceed sequentially to three phases: proliferation, ECM maturation, and mineralization^46^. It takes 16 to 30 days for cells to proceed from proliferation to mineralization; this time varies according to their environment, as shown in in vitro studies^46,47^. Since bone resorption begins soon after bone damage, proceeding with this process as early as possible is essential. In this study, cell proliferation assays (Fig. 8e,f) revealed that the number of MC3T3-E1 cells on CPEEK-AN was approximately twice that of CPEEK 1 week after seeding and, at 3 weeks, was close to each other. This observation indicates that the proliferation phase of cells on CPEEK-AN was quick, reached a confluent state, and smoothly transferred to the next phase; contrarily, the phase for CPEEK dragged on for approximately 3 weeks. Consequently, Ca deposition was superior for CPEEK-AN compared to CPEEK, and collagen and GAG were secreted in higher amounts for AN-treatment than non-AN-treatment (Fig. 9b–d). Previous studies demonstrated that constituents of the mature ECM suppress the osteoclast in many ways^28^. For example, the full length of type I collagen suppresses osteoclast formation, and biglycan, consisting of GAG chains, weakens the osteoclast precursor’s ability to form TRAP-positive multinucleated cells^28,48^. Furthermore, GAG itself has the function of avoiding collagen disruption^49^ under the acidic condition where osteoclast works^50^. Although such advantages for CPEEK-AN were not valid for the ALP activity; these results may serve as a clue for resolving the difference between the ossification process on both substances during the 4–8 week period in the in vivo study. Since ALP is known as an early marker of ossification, it may have already been low at the time of 3-week results^12^.

It is presumed that the bone-bonding strength improvement was elicited by integrating CPEEK and the new bone and a better consolidation process over time. However, a few limitations should be considered: this integration was only observed with SEM, and evaluating all surfaces of each substrate was difficult. In addition, these results were only two time-point snapshots of the remodeling process, which takes a long time.

## Conclusions

The AN-treatment improves bone-bonding strength, and this may have resulted from the direct bonding with the new bone and efficient bone formation process against bone resorption. AN-treatment takes advantage of sulfonation and ACP application and generates a synergetic effect on CPEEK.

## Experimental section

### Substrates

#### Substrate preparation

In vivo studies were conducted on plates of 50C-PEEK (TECAPEEK CM CF50, carbon fiber: 50 wt%, Ensinger, Nufringen, Germany) with dimensions of 15 mm × 10 mm × 2 mm; in vitro studies on disks of the same size with dimensions of 15 mm diameter × 2 mm were performed on 24-well tissue culture plates. Surfaces of substrates were polished using #400 and #1200 SiC abrasive paper. They were air-dried at room temperature after being washed ultrasonically in acetone, ethanol, and distilled water each for 10 min. They were labeled as “CPEEK” groups (Fig. 1).

#### Surface treatment

AN-treatment was performed as reported in our previous study^23^. This was performed as shown in Fig. 2. For sulfonation, PEEK substrates were first soaked in 98 wt% H_2_SO_4_ (Hayashi Pure Chemical., Ltd., Osaka., Japan) twice for 2 s, washed with distilled water, and air-dried at room temperature. Subsequently, the samples were exposed to glow discharge in an O_2_ atmosphere at 200 W (Kyoto Teisan K.K., Kyoto, Japan) for 4 min to get the surface hydrophilicity. Finally, to precipitate apatite nuclei on the substrate’s surface, it was immersed in modified-SBF (Table 1) quickly after oxygen plasma treatment and placed in an incubator at 70.0 °C for 24 h. These samples were labeled as “CPEEK-AN” groups (Fig. 1).

#### Surface characteristics

We analyzed the surface of each sample by field emission SEM (JEOL, Tokyo, Japan) and XPS (JPS-9010TRX, JEOL, Tokyo, Japan) using Mg-Kα radiation at 10 kV and 10 mA. The average pore size was determined with the software CTAn (Skyscan, Bruker, MA).

#### Evaluation of the apatite-forming ability

To assess the apatite-forming ability, each sample described above (CPEEK, CPEEK after sulfonation, and CPEEK-AN) was immersed in the SBF at pH 7.40, 36.5 °C for 1 day. After immersing in SBF, the samples were washed with distilled water, air-dried, and observed using SEM (Fig. S1). Prior to animal experiments, an apatite-forming ability test using SBF was performed to determine whether these substrates had bioactivity.

### In vivo study

#### Animals

The present study was approved by the Animal Research Committee, Graduate Scholl of Medicine, Kyoto University, Japan (Approval number; Med Kyo 21253). All methods in the present study were carried out in accordance with relevant guidelines and regulations. All methods were reported in accordance with the ARRIVE guidelines (https://arriveguidelines.org). Sixteen male Japanese white rabbits (15 weeks old) weighing between 2.8 and 3.3 kg were used, and the operation was done on two legs of each rabbit. In each leg, either of the treatment types of substrate (CPEEK or CPEEK-AN) was implanted. After the rabbits were sacrificed at two-time points, 4 and 8 weeks postoperatively (8 rabbits at each time point), all legs with any substrates were assessed using micro-computed tomography (Skyscan, Bruker, MA). Subsequently, half of them were biomechanically evaluated to determine the chemical bonding strength between substrates and bone, and the other half were histologically evaluated. Apart from them, a rabbit was used for the histological assessment of new bone with TRAP and ALP co-staining. Each leg was assigned to each substrate and sacrificed 4 weeks after implantation.

#### Surgical procedure

Rabbits were anesthetized by intravenous injection of thiopental sodium (30 mg kg^−1^), inhaled isoflurane, and local administration of 1% lidocaine solution. A 3-cm longitudinal skin incision was made on the medial side of the proximal tibia. The fascia and the periosteum were incised and retracted to expose the tibial cortex. After, a slit-like perforation of the same size as the substrates was made using a dental burr from the medial to the lateral cortex parallel to the longitudinal axis of the tibia (Fig. 5a). At 4 and 8 weeks postoperatively, each rabbit was sacrificed with an overdose of intravenous thiopental sodium for biomechanical testing and histological evaluation. Following euthanasia, segments of the proximal tibia containing the implanted samples were cut at the proximal and distal edges of the implant to make the blocks for the following experiments (Fig. 5b).

#### Bone-bonding strength by the detaching test

The detachment test was performed just after taking µ-CT within a few hours from explantation to evaluate the planar bone-bonding strength of each sample^10,11,32^. This test was supposed to simulate the actual failure in the human body caused by shearing power between the new bones and implant. Subsequently, traction was applied vertically to the implant surface at 35 mm min^−1^ using an Instron-type autograph (model 1011; Aikoh Engineering, Japan) (Fig. 5c). Lastly, detachment failure load was measured when the sample plate detached from the bone. If the plate detached before the test, the failure load was defined as 0 N.

#### Radiological evaluation with μ-CT

The block of each sample after harvesting was evaluated using µ-CT scanning with a slice thickness of 0.01 mm (Fig. 5e). The volume in intramedullary areas, which takes 1-mm width from the surface of the substrate (Fig. 5f), is defined as TV. The volume of new bone in this area and its threshold value of > 0.4 g cm^−3^ was defined as BV. Its BMD, BV/TV, Trab.Th, and trabecular surface were calculated using the application (CTAn, Bruker).

#### Histological evaluation

Immediately after CT evaluation, specimens were fixed in phosphate-buffered 10% formalin for 7 days, dehydrated in 70%, 80%, 90%, 99%, and 100% [v/v] ethanol for 3 days at each concentration, and embedded in polyester resin. After, section (1 mm) were cut with a band saw (BS-3000CP; Exakt Apparatebau GmbH, Germany) perpendicular to the tibial axis and ground to a thickness of 100–150 mm using a Micro-grinding MG-4000 (Exakt Apparatebau GmbH) with continuous abrasive papers (#400, #800, #1200, #2000, and #4000). Subsequently, each section was stained with Stevenel’s blue/van Gieson’s picrofuchsin to stain calcified bone, bright red and soft tissue, blue (Fig. 5k). After that, the sections were analyzed digital microscope (DSX 500; Olympus Corporation, Tokyo, Japan) and subjected to quantitative histomorphometry to determine the amount of direct BIC using Image J (National Institutes of Health, USA) as shown in Fig. 5l. Four slices were obtained from each extracted block, and the average value was used for the assessment.

#### The interfacial evaluation between new bone and substrates

In addition to µCT and histology (Fig. 6), SEM (JSM-7900F; JEOL, Tokyo, Japan) was used to assess the bone-implant interface with a similar sample embedded in polyester resin as described above in histological assessment. Three images by each device were compared, as shown in Fig. 7a,b, indicating similar portions were compared. Additionally, higher magnification images were obtained for the interfacial assessment with SEM (Fig. 7c,d). For further observation, an EDS mapping was performed to determine the interface thickness and composition, and line analysis was performed when direct bonding was likely to be obtained (Fig. 7e).

#### TRAP and ALP co-staining assay for new bones

The harvested block from another rabbit at 4 weeks after implantation was used. The paraffin-embedded tissues were decalcified, and cut into 4 µm sections, and stained with a TRAP/ALP staining KIT (Wako Pure Chemical Industries, Osaka, Japan) for histological assessment of osteoclasts and osteoblasts for new bone formation (Fig. 7f).

### In vitro study

Murine calvarial osteoblast cell lines MC3T3-E1 (99072810) were purchased from KOC Co., LTD (Kyoto, Japan), and used for all in vitro studies. They were seeded on the disk-shaped PEEK substrate at 2 × 10^4^ cells/substrate densities in 24-well plates and incubated at 37 ℃. α-MEM (Gibco, USA) with 10 wt% fetal bovine serum and 1 wt% penicillin/streptomycin was donated as a growth culture medium. Conversely, the osteogenic culture medium contained 2 wt% β-glycerophosphate, 0.2 wt% hydrocortisone, and 1 wt% ascorbic acid (Osteoblast-Inducer Reagent. Takara Bio, Tokyo, Japan) added to the growth culture medium. Considering MC3T3-E1 cell has a proliferation phase that covers 4–10 days of culture period followed by bone matrix formation and mineralization^46,47^, growth culture media was switched to osteogenic a week after seeding. Each medium was changed every third day. After incubation for the required period, the following experiments were performed:

#### Cell adhesion on substrates

After 1-day culture in growth culture media, each substrate was washed with phosphate-buffered saline (PBS) and fixed with 2.5% glutaraldehyde for 2 h. After, the substrates were dehydrated in serial concentrations of ethanol (50%, 70%, 90%, 99%, 100%, and 100% [v/v]) for 10 min at each concentration. Subsequently, the substrates were soaked in 50% hexamethyldisilazane (HMDS) (Sigma-Aldrich) with 50% ethanol for 10 min and then soaked in 100% HMDS for 20 min in sequence. All the surfaces of the PEEK plates were coated with platinum and then examined by SEM (Fig. 8a).

#### Cell migration after seeding

Stained cell tracking was performed with time-lapse imaging by the microscope and software (BZ-X800, Keyence, Osaka, Japan) to evaluate the individual cell motion after seeding on each substrate. The nuclei of cells were fluorescently labeled with carboxyfluorescein succinimidyl ester, combined with proteins within cells 3 h after seeding, and incubated for 24 h with the surrounding temperature and CO_2_ concentration maintained at 37 °C and 5% in growth culture media. In addition, 15 randomly chosen nuclei of cells on each CPEEK and CPEEK-AN were tracked, and their locations were recorded every 12 min. Each tracking data was used to visualize the migration of dyed cells rectilinear chart (Fig. 8b–d) and analyze total migration length, maximum velocity defined by maximum migration length in 12 min, and the distance between the first and final place (Figs. S3–S5).

#### Cell proliferation on substrates

Cell proliferation was spectrometrically evaluated by the CCK-8 assay (Dojindo, Kumamoto, Japan), which used WST-8 reduction by dehydrogenases in cells to give formazan dye (Fig. 8e). This assay kit was used for the assay of cell proliferation 1, 2 and, 3 weeks after seeding. At each time-point, the medium was refreshed with PBS containing 10% CCK-8 and incubated at 37 °C for 2 h. After that, the formazan product was quantified by absorbance at 450 nm using a microplate reader (iMarkTM Micro-plate Absorbance Reader, BIO-RAD Laboratories, Hercules, California). Additionally, cell proliferation was evaluated by counting the cell numbers on each surface of CPEEK or CPEEK-AN 1, 2, and 3 weeks after seeding (Fig. 8f). After, the nuclei of cells were stained with DAPI, and they were observed and counted automatically on the overall substrates using the fluorescence microscope and installed analyzer software (BZ-X800).

#### ALP activity

ALP activity was quantified using an ALP assay kit (LabAssay ALP, FUJIFILM Wako, Japan). After 3 weeks of culture, scaffolds were washed thrice with PBS, and the cells were harvested via trypsinization, centrifuged at 1500 rpm for 5 min, lysed using 0.1% Triton X-100, and incubated for 30 min at 37 °C. We confirmed that there were no insolubles after pipetting. Optical density was recorded at 405 nm. The results normalized the total intracellular protein content determined by the bicinchoninic acid assay (Takara BCA Protein Assay Kit, Takara bio) (Fig. 9a).

#### Evaluation of ECM

Collagen secretion was evaluated by the Sirius red staining assay for visualization and quantification 3 weeks after seeding (Fig. 9b). The cells were fixed with 4% paraformaldehyde for 30 min and stained with a picric acid solution (Picro-Sirius Red Stain Kit, ScyTek, Ut, USA) for 2 h. The unbound stain was removed by rinsing with 0.1 M acetic acid. After drying, the staining results were observed and photographed (Fig. 9e). Stained collagen fibers were eluted in a solution composed of 0.2 M NaOH with methanol at a ratio of 1:1. Lastly, absorbance was recorded at 570 nm on the microplate reader.

The dimethyl methylene blue (DMMB) assay (Blyscan Sulfated Glycosaminoglycan assay kit, Biocolor, United Kingdom) was used to quantify the amount of sulfated glycosaminoglycan (sGAG) on the surface of samples Fig. 9c. The procedure was performed following the general protocol of the kit. After 3 weeks of culture, DMMB dye reagents were added to samples lysed in deionized water. Subsequently, after draining from the mixture, the insoluble sGAG-dye complex was dissolved by mixing with a dissociation reagent, including sodium salt, of an anionic surfactant. When all of the bound dye had been dissolved, the mixture was centrifuged at 12,000 rpm for 5 min to remove the form completely, and the absorbance of the mixture was recorded at 650 nm. Finally, an aliquot of 0–10 mg mL^−1^ standards was prepared using a sterile solution of bovine tracheal chondroitin 4-sulfate.

Ca deposition, which expresses ECM mineralization, was evaluated by alizarin red S staining (Fig. 9d). After 3 weeks of culture, the cells on the surfaces were fixed with 75% ethanol for 1 h and stained with 1% alizarin red S solution (Sigma-Aldrich, St. Louis, MO) at room temperature for 30 min. The unbound stain was repeatedly removed with distilled water. After, Ca nodules on the substrates were observed and photographed (Fig. 9f). For quantitation, the bound stains were eluted with 10% cetylpyridinium chloride in 10 mM sodium phosphate; the optical density was measured at 570 nm. CPEEK-AN originally had a certain amount of Ca, and CPEEK-AN, which has no cells, was analyzed as a control.

#### Statistical significance analysis

All graphs show the individual raw value and standard deviation, and statistical significance was determined using the JMP (Ver 15.1.0, SAS Institute, Cary, NC, USA) statistical analysis tool. A two-tailed Student’s t-test was used when only two groups were being compared, and > three groups were analyzed by one-way ANOVA followed by Tukey’s honest significant difference (HSD) test.

* and **indicate statistically significant difference when directly compared to each respective group with *p < 0.05, **p < 0.01.

## Supplementary Information

Supplementary Information.

Supplementary Legends.

Supplementary Figure 1.

## Data Availability

The raw data of this manuscript are available as Supplemental Material.

## References

[1] Kurtz SM, Devine JN (2007). PEEK biomaterials in trauma, orthopedic, and spinal implants. Biomaterials.

[2] de Jong JJA (2017). Distal radius plate of CFR-PEEK has minimal effect compared to titanium plates on bone parameters in high-resolution peripheral quantitative computed tomography: A pilot study. BMC Med. Imaging.

[3] Sacchetti F (2020). Carbon/PEEK nails: A case–control study of 22 cases. Eur. J. Orthop. Surg. Traumatol..

[4] Chua CYX (2021). Carbon fiber reinforced polymers for implantable medical devices. Biomaterials.

[5] *Ensinger Special Polymers Inc., TECAPEEK® CM XP111 BLACK*. https://www.ensingerspi.com/compound.cfm?page=compound&compound=XP-111 (2021) (Accessed 22 October 2021).

[6] *Mitsubishi Chemical Advanced Materials Ketron® 1000 PEEK, Extruded Unfilled Polyetherether Ketone (ASTM Product Data Sheet)*. http://qepp.matweb.com/search/DataSheet.aspx?Bassnum=P1SM12A (Accessed 22 October 2021).

[7] Najeeb S, Zafar MS, Khurshid Z, Siddiqui F (2016). Applications of polyetheretherketone (PEEK) in oral implantology and prosthodontics. J. Prosthodont. Res..

[8] Lee WT, Koak JY, Lim YJ, Kim SK, Kwon HB, Kim MJ (2012). Stress shielding and fatigue limits of poly-ether-ether-ketone dental implants. J. Biomed. Mater. Res. B Appl. Biomater..

[9] Evans NT (2015). High-strength, surface-porous polyether-ether-ketone for load-bearing orthopedic implants. Acta Biomater..

[10] Shimizu T (2016). Bioactivity of sol-gel-derived TiO2 coating on polyetheretherketone: In vitro and in vivo studies. Acta Biomater..

[11] Masamoto K (2019). In vivo and in vitro bioactivity of a “precursor of apatite” treatment on polyetheretherketone. Acta Biomater..

[12] Torstrick FB (2018). Porous PEEK improves the bone-implant interface compared to plasma-sprayed titanium coating on PEEK. Biomaterials.

[13] Ma R (2020). Effects of different sulfonation times and post-treatment methods on the characterization and cytocompatibility of sulfonated PEEK. J. Biomater. Appl..

[14] Wang W, Luo CJ, Huang J, Edirisinghe M (2019). PEEK surface modification by fast ambient-temperature sulfonation for bone implant applications. J. R. Soc. Interface.

[15] Zhao Y (2013). Cytocompatibility, osseointegration, and bioactivity of three-dimensional porous and nanostructured network on polyetheretherketone. Biomaterials.

[16] Liu W (2019). A surface-engineered polyetheretherketone biomaterial implant with direct and immunoregulatory antibacterial activity against methicillin-resistant *Staphylococcus aureus*. Biomaterials.

[17] Combes C, Rey C (2010). Amorphous calcium phosphates: Synthesis, properties and uses in biomaterials. Acta Biomater..

[18] Zhao J, Liu Y, Sun WB, Zhang H (2011). Amorphous calcium phosphate and its application in dentistry. Chem. Cent. J..

[19] Albertini M (2015). Advances in surfaces and osseointegration in implantology. Biomimetic surfaces. Med. Oral Patol. Oral y Cir. Bucal.

[20] Hickner MA, Ghassemi H, Kim YS, Einsla BR, McGrath JE (2004). Alternative polymer systems for proton exchange membranes (PEMs). Chem. Rev..

[21] Cheng Q (2019). Regulation of surface micro/nano structure and composition of polyetheretherketone and their influence on the behavior of MC3T3-E1 pre-osteoblasts. J. Mater. Chem. B.

[22] Christoffersen J, Christoffersen MR, Kibalczyc W, Andersen FA (1989). A contribution to the understanding of the formation of calcium phosphates. J. Cryst. Growth.

[23] Yamane Y, Yabutsuka T, Takaoka Y, Ishizaki C, Takai S (2021). Surface modification of carbon fiber-polyetheretherketone composite to impart bioactivity by using apatite nuclei. Materials (Basel).

[24] Yabutsuka T, Fukushima K, Hiruta T, Takai S, Yao T (2017). Effect of pores formation process and oxygen plasma treatment to hydroxyapatite formation on bioactive PEEK prepared by incorporation of precursor of apatite. Mater. Sci. Eng. C.

[25] Yabutsuka T, Fukushima K, Hiruta T, Takai S, Yao T (2018). Fabrication of bioactive fiber-reinforced PEEK and MXD6 by incorporation of precursor of apatite. J. Biomed. Mater. Res. B Appl. Biomater..

[26] Harris WH, Kwong LM (1993). Autopsy studies of the bone-cement interface in well-fixed cemented total hip arthroplasties. J. Arthroplasty.

[27] Mukka SS (2017). Osteoclasts in periprosthetic osteolysis: The charnley arthroplasty revisited. J. Arthroplasty.

[28] Lin X, Patil S, Gao YG, Qian A (2020). The bone extracellular matrix in bone formation and regeneration. Front. Pharmacol..

[29] Kokubo T, Takadama H (2006). How useful is SBF in predicting in vivo bone bioactivity?. Biomaterials.

[30] Hench LL, Splinter RJ, Allen WC, Greenlee TK (1971). Bonding mechanisms at the interface of ceramic prosthetic materials. J. Bone Miner. Res..

[31] Torstrick FB (2020). Effects of surface topography and chemistry on polyether-ether-ketone (PEEK) and titanium osseointegration. Spine (Phila).

[32] Taniguchi N (2016). Effect of pore size on bone ingrowth into porous titanium implants fabricated by additive manufacturing: An in vivo experiment. Mater. Sci. Eng. C.

[33] Otsuki B, Takemoto M, Fujibayashi S, Neo M (2006). Pore throat size and connectivity determine bone and tissue ingrowth into porous implants: Three-dimensional micro-CT based structural analyses of porous bioactive titanium implants. Biomaterials.

[34] Karageorgiou V, Kaplan D (2005). Porosity of 3D biomaterial scaffolds and osteogenesis. Biomaterials.

[35] Rial R, Liu Z, Messina P, Ruso JM (2022). Role of nanostructured materials in hard tissue engineering. Adv. Colloid Interface Sci..

[36] Tresguerres FGF (2020). The osteocyte: A multifunctional cell within the bone. Ann. Anat..

[37] Hernandez CJ, Majeska RJ, Schaffler MB (2004). Osteocyte density in woven bone. Bone.

[38] Qiao W (2021). Sequential activation of heterogeneous macrophage phenotypes is essential for biomaterials-induced bone regeneration. Biomaterials.

[39] Wolff J (1892). Das Gesetz der Transformation der Knochen.

[40] Liu C (2018). Effects of mechanical loading on cortical defect repair using a novel mechanobiological model of bone healing. Bone.

[41] Wang L, You X, Zhang L, Zhang C, Zou W (2022). Mechanical regulation of bone remodeling. Bone Res..

[42] Park HC (2022). Effect of hydroxyapatite nanoparticles and nitrogen plasma treatment on osteoblast biological behaviors of 3D-printed HDPE scaffold for bone tissue regeneration applications. Materials.

[43] Pulyala P (2017). In-vitro cell adhesion and proliferation of adipose derived stem cell on hydroxyapatite composite surfaces. Mater. Sci. Eng. C.

[44] Matsugaki A, Aramoto G, Nakano T (2012). The alignment of MC3T3-E1 osteoblasts on steps of slip traces introduced by dislocation motion. Biomaterials.

[45] Roseti L (2017). Scaffolds for bone tissue engineering: State of the art and new perspectives. Mater. Sci. Eng. C.

[46] Tevlek A, Odabas S, Çelik E, Aydin HM (2018). Preparation of MC3T3-E1 cell sheets through short-term osteogenic medium application. Artif. Cells Nanomed. Biotechnol..

[47] Choi J, Song K, Park R, Kim I, Sohn K (1996). Expression patterns of bone-related proteins during osteoblastic differentiation in MC3T3-El cells. J. Cell Biochem..

[48] Boraschi-Diaz I (2018). Collagen type I degradation fragments act through the collagen receptor LAIR-1 to provide a negative feedback for osteoclast formation. Bone.

[49] Kram V, Kilts TM, Bhattacharyya N, Li L, Young MF (2017). Small leucine rich proteoglycans, a novel link to osteoclastogenesis. Sci. Rep..

[50] Long F (2012). Building strong bones: Molecular regulation of the osteoblast lineage. Nat. Rev. Mol. Cell Biol..

