# Supplementary material for: Synergistic effect of sulfonation followed by precipitation of amorphous calcium phosphate on the bone-bonding strength of carbon fiber reinforced polyetheretherketone
Source: Sci Rep. 2023 Jan 25;13:1443. doi: 10.1038/s41598-023-28701-1 (PMC9876887; doi:10.1038/s41598-023-28701-1)
Supplement: Supplementary file 2 — Supplementary Legends. [file 41598_2023_28701_MOESM2_ESM.docx]

Supplemental figure

(1) SEM imaging of apatite-forming ability test for each substrate, and EDX profile of the surface of CPEEK-AN before and 1 day after test. (2) The relationship between bone surface / bone volume and trabecular thickness. The evaluation of the cell migration after seeding on substrates for (3) total migration length, (4) maximum velocity, and (5) distance from the starting points.
