# Supplementary material for: Synergistic effect of sulfonation followed by precipitation of amorphous calcium phosphate on the bone-bonding strength of carbon fiber reinforced polyetheretherketone
Source: Sci Rep. 2023 Jan 25;13:1443. doi: 10.1038/s41598-023-28701-1 (PMC9876887; doi:10.1038/s41598-023-28701-1)
Supplement: Supplementary file 3 — Supplementary Figure 1. [file 41598_2023_28701_MOESM3_ESM.pptx]

## Slide 1
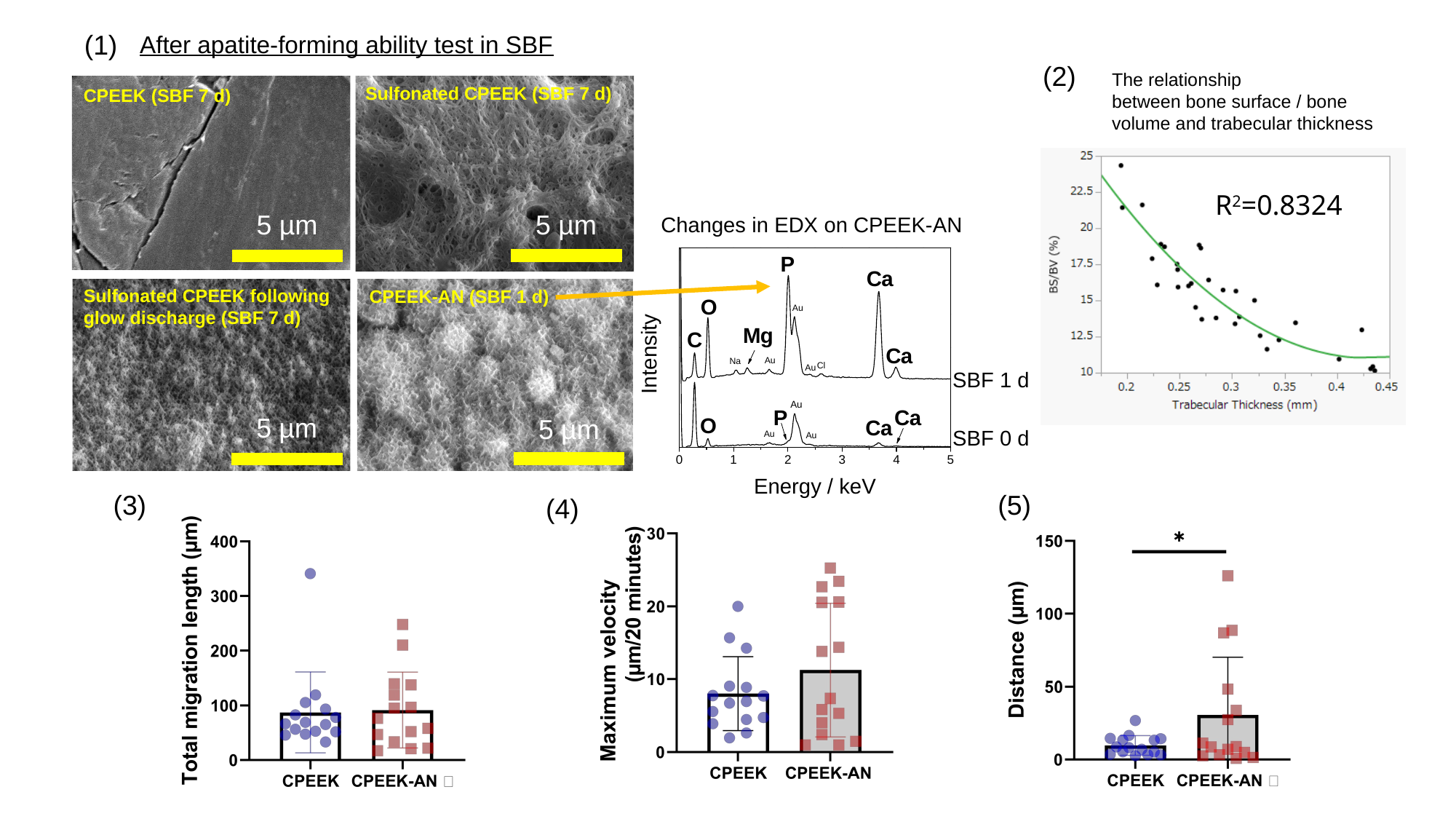

(1)
After apatite-forming ability test in SBF
(2)
The relationship
between bone surface / bone volume and trabecular thickness
Sulfonated CPEEK (SBF 7 d)
CPEEK (SBF 7 d)
R2=0.8324
5 µm
5 µm
Changes in EDX on CPEEK-AN
Sulfonated CPEEK following glow discharge (SBF 7 d)
CPEEK-AN (SBF 1 d)
SBF 1 d
5 µm
5 µm
SBF 0 d
(3)
(5)
(4)

## Slide 2
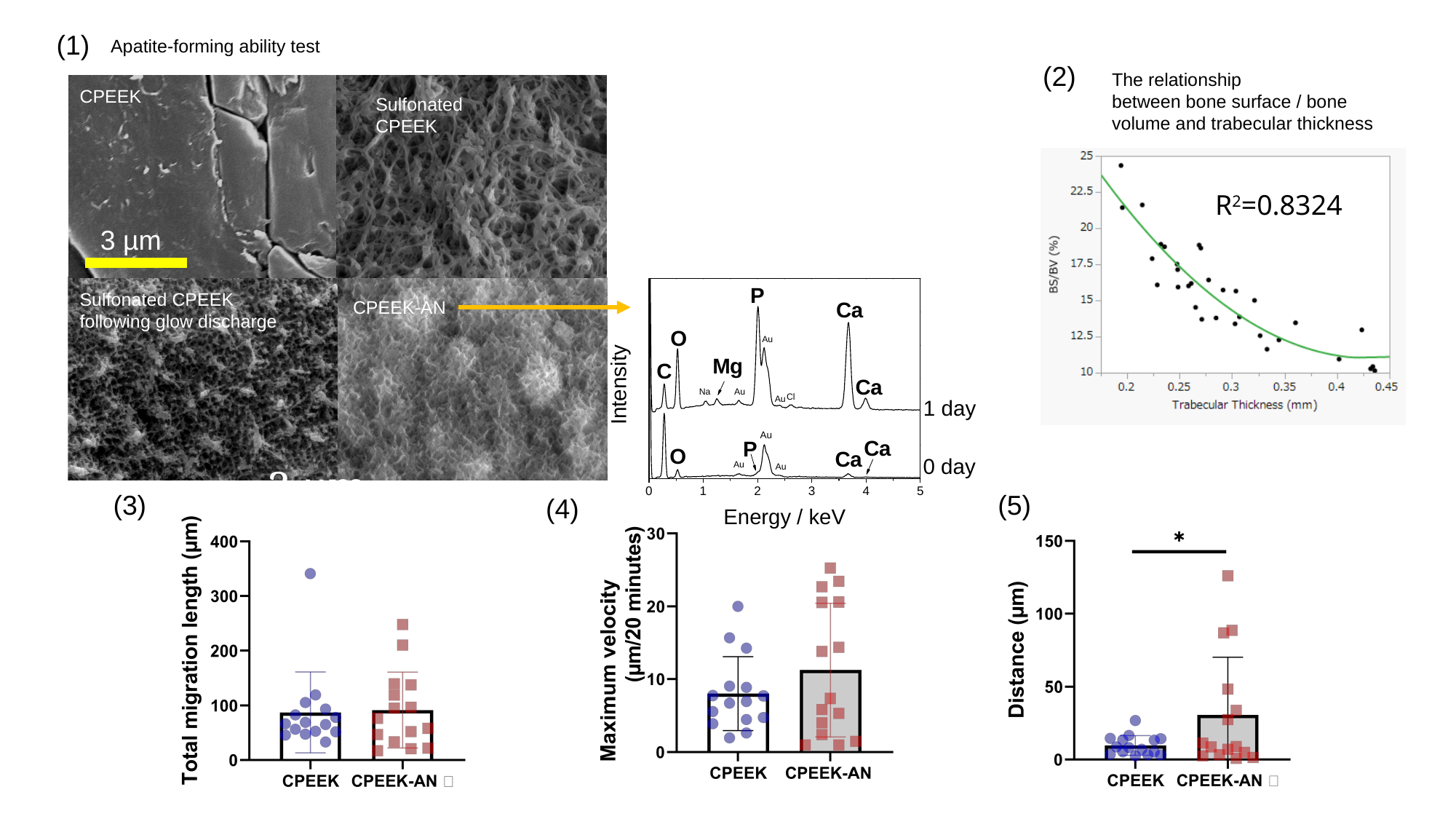

(1)
Apatite-forming ability test
(2)
The relationship
between bone surface / bone volume and trabecular thickness
CPEEK
Sulfonated CPEEK
R2=0.8324
3 µm
Sulfonated CPEEK
following glow discharge
CPEEK-AN
(3)
(5)
3 µm
(4)
